# Two Cases of Disease of the Heart

**Published:** 1826-08

**Authors:** 

**Affiliations:** the Middlesex Hospital


### DISEASE OF THE HEART.

*Two Cases of Disease of the Heart.* Treated at the Middlesex Hospital, by Dr. Macmichael.

### I. *Case in which Tubercles were found in the Pericardium and Cavities of the Heart, as well as in the Lungs.*

J. Burrows, aged thirty-five; admitted April 4th, died June 2d, 1826. This man, during life, laboured under symptoms indicative of disease on the right side of the heart. He was originally admitted into the hospital for colica pictonum, and, upon the subsidence of this affection, the following symptoms claimed the attention of his physician.

The upper half of the body became œdematous; the countenance was livid, and occasionally of a purple colour; the breathing was unnatural, and at times even difficult; the pulse did not deviate much from health. There was considerable anxiety depicted in the countenance,—that peculiar expression so characteristic of disease of the heart, or large vessels connected with it. In short, there was evidently an obstruction offered to the free transmission of the venous blood on the right side of the heart, causing an accumulation in that part, and turgescence in the large venous trunks. The serous infiltration into the cellular membrane, producing œdema of the upper part of the body, appeared to be the effect of this obstruction to the venous circulation.

He continued in this state for several weeks. The little sleep he had was disturbed: medicines appeared to have but little effect, even as palliatives; the symptoms became more severe; the lividness of the surface, and the œdema, increased; and he at length sunk, exhausted and worn out by the long continuance of his disease.

The preceding brief sketch will serve to connect the symptoms with the morbid appearances.

*Dissection.—Thorax:* The lungs were completely disorganised: the parenchymatous structure was thickly studded with white tubercles, in different stages of development. Here and there this process appeared to be so far advanced as to have removed the tubercles themselves; the cysts which they had occupied now containing pus of a scrofulous character, and forming abscesses of different dimensions. Other parts of the lungs were found to contain a ligamentous structure, which was cut through with some difficulty. So numerous were these different morbid changes throughout the substance of the lungs, so dense was their structure, and so universally adherent were they to the surrounding parts, as to cause great difficulty in removing them from the chest.

The pericardium contained tubercles, apparently of the same structure: these were situated around the base of the heart. At the angle formed by the reflection of the pericardium over the surface of the heart, they were covered by the delicate serous membrane. On opening the heart and large vessels, this morbid growth was found to encroach even upon their cavities. A large tubercle, situated at the union of the two cavæ, and others surrounding the inferior cava, just after its passage through the diaphragm, had so straightened these vessels, that it was with difficulty that the little finger could be made to pass the obstruction. A similar morbid growth was observed in the right auricle, forming a considerable projection: this occupied the septum auriculorum, and thus implicated also the left auricle.

This dissection satisfactorily accounts for the symptoms of venous congestion on the right side of the heart; and it is not at all improbable that death was caused by the long-continued circulation of imperfectly arterialised blood : indeed, it is only astonishing how life could have been so long supported with such a diseased state of lungs. It is worthy of remark, that this patient never had cough, expectoration, or any other symptom of phthisis.

Although Boerhaave is supposed to have described tubercles of the serous membranes, and Bichat, with De Haen and others, have given more accurate descriptions of this disease, still (with the exception of the latter author,) Dr. Baillie appears first to have noticed tubercles of the pericardium. He states that they are of very rare occurrence, and that he has only met with one instance of their formation in that membrane. Dr. Baron, in his work on Tubercles of the Serous Membranes, has not treated of this disease in the pericardium.

The above case was at first supposed to be an example of the disease described by Dr. Baillie; but, on making a more accurate examination, it evidently appeared to be merely a propagation of the tubercles from the lungs; for it was singular that not one of these formations was in an insulated state, but they were found to be invariably connected with tubercles arising from the lungs. Some of them appeared to be the bronchial lymphatic glands in a diseased state. There was no inflammation of the heart or pericardium. Proofs, however, of the independence of tubercles on inflammation for their production, have now ceased to be necessary. If a doubt on this subject does still remain, we have here an instance of these formations in the heart itself, still without any symptoms of inflammation during life, or marks of the existence of such a state exhibited after death.

*Case of Enlargement of the Heart, (the presence of which was not manifested by the symptoms,) and of Acute Hepatitis.*

April 7th, 1826.—James Fisher, aged thirty-six, was admitted, on the 5th, with slight rheumatic symptoms: indeed, so slight were they as to lead to some doubt of the necessity of his becoming an *in-patient.* The bowels were opened, and some camphor mixture, with colchicum, given every six hours. Next day, his rheumatism was better, but some yellowness of the skin was observed. The tongue was clean, and the pulse natural. During the night of the 6th, the apothecary was suddenly called to him: he found him complaining of pain at the scrobiculus cordis and right hypochondriac region, which was increased on pressure; his breathing was oppressed. These symptoms continued to increase, and within four hours had acquired great intensity. The pain was very severe, and much aggravated by pressure; the respiration was difficult, with pain behind the right scapula; in short, the patient evidently laboured under an attack of acute hepatitis. Twenty-four ounces of blood were taken from the arm, from which he experienced great relief, and soon after fell asleep. The blood drawn was cupped, and its surface covered with a buffy coat of a yellow colour.

About ten o'clock this morning, he died suddenly, having been seen about a quarter of an hour before, at which time he appeared to be much better, and expressed himself to that effect.

*Dissection.*—The skin of the whole body was of a deep yellow colour.—*Abdomen*: The liver was much enlarged, and of a purple colour. On making a cut into its substance, it exhibited a granulated and firm texture, arising from interstitial deposition. The vessels appeared to be gorged with blood; the peritoneal covering was perfectly natural. The viscus had evidently been some time diseased, but appeared to have recently taken on general inflammatory action.

*Thorax:* The heart was of unusual size, and weighed two and a half pounds: a great increase, when it is remembered that the human heart, in a state of health, weighs only a few ounces.* The coronary arteries were thicker in their coats than natural, but there were no traces of ossification. The enlargement of the heart in this case presented some peculiarities. The right auricle and ventricle were much dilated, and the communication between these two cavities had suffered with the general enlargement, so much that the valves, in consequence, must have been incapable of duly performing their office. The walls of this ventricle were softened; the muscular parietes were pale and degenerated: in short, the right auricle and ventricle were as one large and flabby pouch, stuffed with a dark coagulum, and their structure corresponded to the softening of the heart described by Portal. The left cavities presented a striking contrast with the above description: the muscular walls of the left ventricle were thicker and more vascular than natural, forming hypertrophy of the left ventricle;† the aortic valves were slightly diseased; but any mechanical obstruction which could have been offered by them was too trifling, and altogether inadequate to the production of this morbidly increased growth of the ventricle.

The lungs were found to be universally adherent to the pleura lining the ribs, and had suffered from a considerable effusion of serum into their substance.

It is remarkable that such extensive disease of the heart should have existed without the occurrence of any symptoms during life indicating its presence. This circumstance countenances the opinion of the late Mr. Burns, who states that symptoms of diseased heart seldom occur, unless inflammation supervenes, or the enlargement be of an active character.

---

### SLOUGHING ULCERATION.

* Lieutaud has described a heart which weighed five pounds.

† When thickening of the parietes of the heart arises from mechanical obstruction to the circulation, it forms (according to some continental writers) a disease different from that which is termed hypertrophy. M. Bertin has shown that hypertrophy may occur in the contracted, natural, and dilated states of the cavities of the heart.

